# Health with Economy: The Position on the South Coast

**Published:** 1922-03

**Authors:** 


### HEALTH WITH ECONOMY.

### THE POSITION ON THE SOUTH COAST.—I. EASTBOURNE.

NOW that the axe is laid to many fruitful branches of the tree of Public Health, it is interesting to ask how the necessity for economy is affecting individual boroughs. Eastbourne, in the persons of the Chairman of its Health Committee, Mr. Alderman Martin, and its Medical Officer of Health, Dr. W. G. Willoughby, is able to answer cheerfully that it had the good fortune to be well ahead of its own health problems before the necessity arose. Consequently its service will be comparatively little hampered in these lean years. The Tuberculosis Hospital was finished in 1914 and opened a few days before war was declared. An open-air school for delicate children has been established, and the maternity home has been very useful for cases that could not be properly nursed in overcrowded houses.

The decision of the Government to reduce the milk grant and to exclude children of from one to five years is regarded in Eastbourne as a mistake. The extra milk was of great value to many of the older children as well as to the infants. So far free meals have not been given officially to nursing mothers instead of milk, but there are various voluntary organisations in the town which give them, particularly those connected with the churches. It is not proposed to give dinners except in cases of absolute necessity; indeed, the Ministry of Health has been asked only for £50 a year for this purpose. The view of the Public Health Committee is that the mothers cannot be persuaded to go out for their dinners, because these are ready just at the time when they are preparing and serving the family midday meal. This was actually tested in connection with a voluntary scheme.

At present there are between 1,300 and 1,400 unemployed. Eastbourne being a health resort, people have been drawn there who are not well and will never be fit for steady work, so a good many of these are unemployable. All health resorts are more or less in the same position. Not long ago a man with tuberculosis was asked why he came to the town. His doctor had told him to go there and try to get work. There is comparatively not much distress, as a substantial dole is given, and public health is not greatly affected, except for a slight rise in infant mortality and a rise in tuberculosis.

Eastbourne has not escaped influenza, but it has not been of severe type. There has been little pneumonia and only half-a-dozen deaths, the average age being over sixty. Following the precedent of the big epidemic three years ago, a ward for influenza was opened in the Infectious Diseases Hospital.

The proposal to increase the age at which a child enters the elementary school from five to six meets with approval. Six is considered quite young enough —from the child's point of view, decidedly so. It is thought that difficulty will be with the mothers, who would have to stop at home or make arrangements to have the children taken care of for the extra year. But though formal education could very well be put off until six, children of from four to six years might go to nursery schools, where they could be looked after and amused by sensible, motherly women—not highly-paid teachers.

The view that it is a waste of public money to admit cases of scarlet fever to hospital, and that, instead, measles, whooping cough and summer diarrhœa might be admitted, is not held at Eastbourne, where, it must be remembered, there are over 2,000 children in boarding schools. If the first case is removed it is almost invariably found that no more cases occur in the same house. When nursed in hospital they do not develop pleurisy or the chronic kidney trouble that might make them, within a few years, an expense to the community. Then, if they were at home the household would have to be isolated and people in the food trades and laundry-women would be kept from work. By this policy the scarlet fever death-rate has been reduced. Measles and summer diarrhœa cause far more deaths than scarlet fever, and given space, the Eastbourne authorities would isolate them also. They are admitted as far as possible, and once or twice whooping cough has been admitted when a ward was empty and could be spared. With regard to summer diarrhœa, there is, as a rule, only one baby in a house, and therefore the disease is less likely than others to spread. While cases are sometimes removed to hospital, they are usually dealt with by the health visitors in the houses.

With regard to sewage disposal, Eastbourne is a modern town, and therefore its sanitation is good. The water-carriage system exists everywhere, except in a few agricultural buildings in outlying districts. The sewage is carried a mile from the end of the town to Langney Point, in Pevensey Bay, where the currents take it away off the foreshore, and it is screened before it goes into the sea. The question of foreshore pollution happily does not arise; it can occur only under exceptional conditions of wind and tide, and, as a matter of fact, there have been only two complaints in 27 years. Eastbourne was the first town to introduce a system of sanitary certificates for houses. The Corporation examine a house free of charge, and give it a certificate as being up to date, and will re-examine after three years. Between 1,000 and 2,000 houses out of 9,000 have these certificates, which are, of course, an excellent advertisement for those which let rooms. The smaller houses do not ask for certificates, though they could have them.

The Corporation has dealt energetically with the housing problem. It has put up 178 houses, and the Ministry of Health has given permission for another 33, which are about to be begun. Had it been allowed it would have built more. In order to deal with overcrowding in the town at the end of the war, the vacated Army huts were taken over and fitted up as dwellings for civilians. At one time there was a hut population of 562. The majority of these huts are still standing and contain about 120 families.

As most of the streets have been built within the last 50 years, they are all of good width, which means that there are no slums; slums and wide streets are incompatible. Eastbourne is one of the two towns in the country that have a Building Surveyor separate from any other department, and its sanitary inspectors are very alert.

The death rate is one of the lowest in the country. The infantile mortality rate for 1921 was 59·8 only, and the total death rate 10·28 per 1000. For a long period preceding the war it did not reach 11 per thousand; in 1914 the total rate was only 9·4, and the average is 10·5. It is a health resort singularly favoured in the matter of sunshine. Last year it was the second town in the United Kingdom in this respect, and the first on the south coast. In three years out of ten it was the first in the United Kingdom. The front of the town is open to the sea, facing south and south-east, and this has its effect upon the record. Eastbourne is a progressive town, with a very high rateable value for its population, and the Council has been able to pay for such desirable things as its own Tuberculosis Hospital and open-air school for delicate children. It has a smallpox hospital, which remains empty, and has had no case since 1902. Since it was built, early in the nineties, it has received only 27 cases.

The water of the town comes from a deep well in the South Downs, about four miles out, at Friston, and is pumped into distributing reservoirs; but is not stored for more than a day or two. The reservoirs are up to date and covered. There are always over 30 gallons a head per day for consumption, and the water is never turned off night or day. Eastbourne has its own baths and swimming baths. It encourages swimming among the elementary school children, giving certificates and holding an annual gala for them. Individual schools have their own galas also.

The recent census showed that the population of Eastbourne has increased 18 per cent. in the ten years—more than any other county borough except three.

